# Characteristics of YouTube Videos in Spanish on How to Prevent COVID-19

**DOI:** 10.3390/ijerph17134671

**Published:** 2020-06-29

**Authors:** Ignacio Hernández-García, Teresa Giménez-Júlvez

**Affiliations:** 1 Department of Preventive Medicine, Lozano Blesa University Clinical Hospital of Zaragoza, Calle San Juan Bosco 15, 50009 Zaragoza, Spain; 2 Department of Preventive Medicine, Miguel Servet University Hospital of Zaragoza, Paseo Isabel la Católica 1, 50009, Zaragoza, Spain; tgimenez@salud.aragon.es

**Keywords:** coronavirus, COVID-19, YouTube, information, prevention, World Health Organization

## Abstract

Objective: To analyze the characteristics of YouTube videos in Spanish on the basic measures to prevent coronavirus disease 2019 (COVID-19). Methods: On 18 March 2020, a search was conducted on YouTube using the terms “Prevencion Coronavirus” and “Prevencion COVID-19”. We studied the associations between the type of authorship and the country of publication with other variables (such as the number of likes and basic measures to prevent COVID-19 according to the World Health Organization, among others) with univariate analysis and a multiple logistic regression model. Results: A total of 129 videos were evaluated; 37.2% were produced in Mexico (25.6%) and Spain (11.6%), and 56.6% were produced by mass media, including television and newspapers. The most frequently reported basic preventive measure was hand washing (71.3%), and the least frequent was not touching the eyes, nose, and mouth (24.0%). Hoaxes (such as eating garlic or citrus to prevent COVID-19) were detected in 15 videos (10.9%). In terms of authorship, papers produced by health professionals had a higher probability of reporting hand hygiene (OR (95% CI) = 4.20 (1.17–15.09)) and respiratory hygiene (OR (95% CI) = 3.05 (1.22–7.62)) as preventive measures. Conclusion: Information from YouTube in Spanish on basic measures to prevent COVID-19 is usually not very complete and differs according to the type of authorship. Our findings make it possible to guide Spanish-speaking users on the characteristics of the videos to be viewed in order to obtain reliable information.

## 1. Introduction

Internet access has increased worldwide in recent years. By the end of 2017, 3.58 billion people were online, equivalent to 48.0% of the global population [1].

YouTube is the second most visited website and the second biggest search engine in the world [2]. In particular, YouTube, with more than one billion users worldwide and more than 100 million videos, is becoming an increasingly important source of health information [1,3], and it has the capacity to influence its users (for example, regarding their vaccination habits [3] or patient decision-making regarding screening and prevention on colorectal cancer [4] or buying a drug [5]). However, it has no policy of filtering videos according to their potency or effectiveness; for this reason, there are many videos online, and while some may be useful, others may be misleading [6].

In this context, infodemiological studies are becoming increasingly necessary, since they can provide valuable insights into health-related behaviors of populations. Infodemiology is the science of distribution and determinants of information on the Internet or in a population, and it has the aim of informing public health and public policy [7]. Examples of infodemiology applications include the identification and monitoring of public-health-relevant publications on the Internet, measuring information diffusion, and analyzing how people search and navigate on the Internet for health-related information as well as how they communicate and share this information [7,8].

During the previous Zika [9] and Ebola [10,11] public health emergencies, YouTube videos on such infections were viewed millions of times [9,10,11]. This extraordinary audience makes YouTube a double-edged sword in times of epidemic crises, because while appropriate YouTube content can benefit health organizations in ensuring that the population properly implements measures to control the spread of the disease, misleading videos can contribute to failure to contain the infection [12].

On 11 March 2020, the World Health Organization (WHO) considered coronavirus disease 2019 (COVID-19) to have a pandemic status [13]. According to the WHO situation reports, as of 18 March 2020, 191,127 cases had been confirmed worldwide, of which 11,178 were in Spain and 701 cases were in 16 Spanish-speaking countries of America [14]. As in other similar situations, people wanted to know what they could do to prevent and treat the disease [15]. Since there is currently no vaccine or specific antiviral treatment, the application of basic preventive measures is essential [16].

The objective of this study was to evaluate the characteristics of the YouTube videos that provide information in Spanish on the basic measures for preventing COVID-19.

## 2. Material and Methods

On 18 March 2020, a cross-sectional study of the data was conducted by entering the terms “Prevencion Coronavirus” and “Prevencion COVID-19” into the YouTube search engine. The videos were sorted according to the number of views, and their full names and URLs were recorded. After applying the exclusion criteria (not available for viewing, language other than Spanish, not providing information on COVID-19, and duplicate video), the 129 most viewed videos were selected. This sample size was estimated from the total number of videos that met the selection criteria (379 videos, after having discarded 39 for being duplicates, 5 for using a language other than Spanish, and 1 for not providing information on COVID-19; all were available for viewing), considering an accuracy level of 5%, an alpha error of 5%, and an expected proportion of finding information on how to prevent COVID-19 according to the WHO of 15% [17].

The information corresponding to the following variables was extracted: date and country of publication, number of views, comments, likes and dislikes, duration, and type of authorship (represents the person or organization that produced the video and was classified into 4 categories: “mass media”, including television and newspapers; “health professionals”, including healthcare professionals, medical centers, or public health official organizations; “individual users”, a lay person’s opinion about the issue; and “others”, videos that did not belong to any other category [3]). In addition, we recorded whether they provided information on the following basic measures to prevent COVID-19 according to the WHO: (a) wash your hands frequently; (b) respiratory hygiene (when coughing or sneezing, cover the mouth and nose with a bent elbow or tissue, and then dispose of the used tissue immediately and wash your hands); (c) social distance (keep at least 1 m away from other people, particularly those who are coughing, sneezing, or have a fever); and (d) avoid touching your eyes, nose, and mouth) [18]. 

One author (I.H.-G.) reviewed and encoded the content of all videos, and a second author (T.G.-J.) reviewed and encoded a subset of 40 randomly selected videos.

A descriptive analysis of the variables was performed, and we studied which variables were associated with the videos providing information on the basic measures to prevent COVID-19. For this purpose, the countries of origin of the videos were categorized according to the type of transmission existing in each country at the time of data collection, as follows: (a) local transmission, locations where the source of infection is within the reporting location; (b) imported cases only, locations where all cases have been acquired outside the location of reporting; and (c) no transmission, countries where no cases have been reported) [14]. 

## 3. Data Analysis

The chi-square test, or Fisher’s exact test, was used for qualitative variables. The magnitude of each association was quantified with the odds ratio (OR) and its 95% confidence interval (CI) obtained with a univariate logistic regression analysis. For quantitative variables, after checking with the Kolmogorov–Smirnov test that none followed a normal distribution, the Mann–Whitney U-test was used. 

A multivariable logistic regression analysis was performed with all variables associated (*p* < 0.05) with the videos providing information on basic measures to prevent COVID-19. The agreement between the two reviewers regarding the basic protective measures in the videos was analyzed using the Kappa index.

All statistical analyses were performed using SPSS v25 (IBM Corp, Chicago, USA) and EpiInfo (Centers for Disease Control and Prevention, Atlanta, USA). As in similar studies, videos publicly available on YouTube were assessed, and no human participants/animals were included. Therefore, Ethics Committee approval was not required for this study [19].

## 4. Results

The oldest video was dated 13 January 2020, and the most recent was dated 18 March 2020. A total of 41.9% of the videos were published on or after 11 March 2020, and 37.2% (48/129) were produced in Mexico or Spain (Table 1). A percentage share of 78.3% of the videos were produced by the mass media (56.6%) and health professionals (21.7%). Only one video was produced by the WHO.

The videos had been viewed 15,589,902 times. The number of views ranged from 10,053 to 1,933,567 (median: 45,284). The number of comments ranged from 0 to 2639 (median: 85). In terms of likes and dislikes, the figures ranged, respectively, from 0 to 48,367 (median: 394) and from 0 to 2711 (median: 32). The median duration was 187 s (range: 30–6485 s). 

There were no discrepancies between the authors regarding the basic protective measures reported in the videos (Kappa = 1). The most frequently reported basic preventive measure was hand washing (71.3%), and the least frequent was not touching the eyes, nose, and mouth (24.0%) (Table 1). 

Hoaxes were detected in 15 videos (10.9%). In particular, seven videos indicated that certain foods (such as garlic, citrus, zinc-containing foods, parsley, ginger tea with curcuma, or fennel tea) or the consumption of multivitamin supplements, magnesium chloride, sodium bicarbonate, water with bicarbonate plus lemon, alkaline water, N-acetyl cysteine tablets, or zinc tablets serve to prevent COVID-19. One video also indicated that “orange juice with kiwi and a spoonful of pollen is more important than hand hygiene to prevent COVID-19”. 

One video indicated that with rising temperatures in the spring, heat will help to control COVID-19. Two videos indicated that natural sunbathing serves to prevent COVID-19; one of them also indicated how hot/cold contrast baths serve to prevent COVID-19.

Five videos referred to conspiracy theories about the origin of the virus (created in a laboratory to be used as a biological weapon or because of the interest of the pharmaceutical industries to make a new treatment or a new vaccine). In addition, one of these videos questioned the usefulness of recommending hand hygiene or respiratory hygiene practices because in Spain and Latin American countries, people will not comply because of their culture/way of being.

Significant differences were detected in the number of video views reporting hand washing as a measure to prevent COVID-19 (median: 49,972; interquartile range: 21,690–122,269) compared to those not reporting such information (median: 29,359; interquartile range: 14,775–70,115) (*p* = 0.039). Significant differences were also detected in the number of comments on the videos reporting respiratory hygiene as a measure to prevent COVID-19 (median: 49.5; interquartile range: 14.75–169.75) compared to those not reporting such information (median: 128; interquartile range: 36–251.5) (*p* = 0.023).

According to the author, those produced by health professionals showed, compared to the rest of the videos, a higher probability of reporting on washing hands frequently (OR (95% CI) = 4.23 (1.19–15.01); *p* = 0.018), respiratory hygiene (OR (95% CI) = 3.39 (1.42–8.14); *p* = 0.005), and avoiding touching the eyes, nose, and mouth (OR (95% CI) = 3.24 (1.32–7.96); *p* = 0.009) as measures to prevent COVID-19. In particular, videos produced by health professionals showed, compared to those made by the mass media, a higher probability of reporting on washing hands frequently, respiratory hygiene, and avoiding touching the face as measures to prevent COVID-19 (Table 2). Moreover, significant differences were detected in the frequency with which the videos produced in countries with local transmission (Spain, the United States of America, Argentina, Colombia, Ecuador, Germany, Chile, Peru, Costa Rica, Dominican Republic, China, the United Kingdom) provided information on washing hands frequently (OR = 2.29) and respiratory hygiene (OR = 2.26) compared with other countries (Table 3). No other associations were found in the univariate analysis.

In the multivariate analysis, the only variable that maintained a significant association with the video reporting on hand hygiene and respiratory hygiene as measures to prevent COVID-19 was the type of authorship. Videos produced by health professionals showed, compared to the rest of the videos, a higher probability of reporting on washing hands frequently (OR (95% CI) = 4.20 (1.17–15.09); *p* = 0.028) and respiratory hygiene (OR (95% CI) = 3.05 (1.22–7.62); *p* = 0.017).

## 5. Discussion

This study is the first to evaluate the characteristics of YouTube videos that provide information specifically in Spanish on the basic measures indicated by the WHO to prevent the transmission of COVID-19. The most frequently reported basic preventive measure was hand washing (71.3%), and the least frequent was not touching the eyes, nose, and mouth (24.0%). Hoaxes were detected in 15 videos (10.9%). 

The videos in this study had accumulated a total of 15,589,902 views. This number, together with the median number of views obtained (45,284), is lower than those recorded in studies that have analyzed the information on YouTube on recent pandemics, such as Ebola, in which the total number and the median number of views in the 100 most viewed videos were 73 million times and 401,162 views, respectively [10]. It is a surprising result, given the greater global spread of COVID-19 than Ebola. Moreover, this is a very worrying finding (given that 483 million people have Spanish as their mother language [20]), and it could indicate that, on the day of the data collection (On 18 March 2020), the Spanish-speaking population had little interest in and concern about how to prevent COVID-19, despite the fact that a week earlier, the WHO had declared COVID-19 to be a pandemic [13]. Another disturbing finding was that three of the four basic prevention measures of the WHO appeared in less than 42% of the videos. It is difficult to control the spread of a virus if people have little information and are not very interested in how it can be prevented. Perhaps all of this may have contributed to the fact that, in Spanish-speaking countries, the number of cases multiplied in just two weeks, which corresponds to the incubation period of COVID-19 [21]. For example, Spain went from 11,178 cases on March 18 to 94,417 cases on 1 April, Chile from 156 to 2738 cases, Ecuador from 58 to 2240 cases, and Peru from 86 to 1065 cases [14,22]. Unfortunately, this trend has been increasing in Latin America, and at the end of May, the WHO declared that Central and South America have emerged as the new epicenter of the coronavirus pandemic [23]. 

In this regard, the presidents of several governments (including those of Peru and Chile) have stated that this pandemic has taught them that the priority must be education [23]. It may also be a lesson to be learned for the management of future pandemics, as using YouTube to educate early represents an opportunity and a means to this goal [2]. Timely education is a fundamental element of any prevention policy; prevention is either reached earlier or not at all [24]. Knowledge regarding infectious diseases is related to the level of adherence to all control measures, which may limit the spread of those diseases [25].

The difficulty of obtaining information on YouTube on the basic measures for the prevention of COVID-19 according to the WHO is congruent with studies by other authors that have described the difficulty of finding measures promoted by the WHO to prevent other infectious diseases, such as influenza, on the Internet [26]. In fact, this limited availability of information was also described by Basch [17], who, after evaluating 100 YouTube videos on COVID-19 (86 in English and 14 in Spanish), stated that the videos reported maintaining social distance, hand washing, and respiratory hygiene as fundamental prevention measures in only 31, 26, and 14 videos, respectively [17]. However, the fact that this study did not provide disaggregated information according to the language of the video limits the validity of making comparisons with our results, given that, as previously discussed in other works, it is common for videos in English to provide information on preventive measures against infectious diseases less frequently than videos in Spanish [27].

Our study is the first to provide data on infodemics related to the prevention of COVID-19 detected specifically in YouTube videos in Spanish. In particular, hoaxes were detected in 10.9% of the videos. Such findings could be used in educational campaigns to correct misinformation about COVID-19 in Spanish. The fight against this pandemic is also a fight against infodemics. Nowadays, misinformation is an important problem; people do not tend to critically assess the information they read [5]. Dispersing misinformation can create agitation; can cause fear, panic shopping, and taking drugs without a medical prescription; and can ultimately diminish preventive measures [5,28]. 

Misinformation on COVID-19 is rife, especially on social media [29]. Studies on this have been done on Twitter and the Internet. In particular, Kouzy [30] analyzed 673 tweets related to COVID-19 and found misinformation in 24.8%. Cuan [5] compared information about the new coronavirus available on 36 websites with information from a medical bibliography, finding that 15 websites gave true information, 16 gave partially true information, and 5 gave false information compared to the medical literature present in PubMed.

The study of misinformation about COVID-19 in YouTube videos has also been carried out, although only from videos in English. Li [31] evaluated 69 YouTube videos in English and detected that 19 (27.5%) contained misinformation. However, this author did not specify examples of misinformation related to the preventive measures of COVID-19 in his publication, a fact that limits the possibility of making detailed comparisons with the findings of this study.

Social media providers are trying to filter out fake news, but this has not stopped the conspiracy theorists, swindlers, and liars on the Internet [5], as we have detected in our study. Scientific information about COVID-19 flows freely in the networks, but it must be accompanied by proper interpretation by the media and Internet users. However, for users with nonmedical education, it is difficult to judge the reliability of health information on the Internet [5]. For these reasons, and in this context, the WHO has recently provided information on its website to counteract various hoaxes about COVID-19, explaining, for example, that exposing yourself to the sun or to temperatures higher than 25 ℃ does not prevent COVID-19, taking a hot bath does not prevent transmission of the new coronavirus disease, and that there is no evidence that eating garlic has protected people from the new coronavirus [32].

In any case, to combat misinformation in social media, more ambitious strategies must be adopted. According to Bastani and Bahrami [33], such strategies would include the following: supervision of online content via regulation setting and the creation of a legal framework, identification of acommunity’s information needs related to COVID-19, participation of specialists and providers of health institutions in generating valid and credible information, and facilitation of the dissemination of evidence-based information [33].

With regard to authorship, the finding that the majority of videos were produced by mass media (60.0%) supports what has been found in previous studies carried out to date on COVID-19 in YouTube videos, in which the mass media were found to have produced 85.0% [17] and 75.4% [34] of the videos evaluated. Given that our work found that, in general, the probability of finding information on the basic preventive measures of COVID-19 is lower in videos produced by mass media, mass media must be urged to assume responsibility for providing correct and complete information and creating understanding among citizens [35]. In addition, since the highest probability of obtaining information on basic prevention measures for COVID-19 was obtained from videos by health professionals, which confirms findings in other studies regarding the reliability of information in the videos of such professionals [2,36], Spanish-speaking users should be encouraged to consult videos produced by health professionals when looking for information on YouTube on how to prevent COVID-19. Furthermore, implementing and evaluating the effectiveness of this behavior could be the subject of future research.

No specific differences were found according to the country of origin of the video. This could be explained by the fact that although the local situation in terms of the type of transmission of COVID-19 was variable [14], the basic prevention measures studied are recommended to be applied worldwide.

Among the limitations of our study, one that stands out is that it is intrinsic to the Internet, since online information is constantly changing, and this type of research is limited to the information available at the specific time it is analyzed [3,9,10,17,27,34]. In addition, the search terms were chosen by the authors on the assumption that a Spanish-speaking user would probably use one of them to perform simple searches on YouTube regarding COVID-19 prevention measures. The number of videos evaluated, although somewhat less than that used by some other authors (142) [37], is greater than that used in most studies of this type [6,9,10,11,17,19,31,34], in which less than 114 videos are usually included. In any case, our sample was sufficient to obtain accurate results (with narrow confidence intervals).

## 6. Conclusions

This work shows that, a week after COVID-19 was considered a pandemic, the information in YouTube videos in Spanish about the basic measures to prevent COVID-19 according to the WHO was incomplete and differed according to the type of authorship. In addition, such videos had rarely been viewed. This represents an alarming finding, given that a key element in controlling the spread of this disease is that people know what to do to prevent it, since people cannot implement disease control measures if they do not know them. This is perhaps a lesson to be learned from this crisis. Thus, for future pandemics, there is an urgent need for early training of the population in basic prevention measures. The key to this is to disseminate information on prevention measures recommended by the WHO more frequently and to promote its consult with particular emphasis on Spanish-speaking users consulting YouTube videos produced by health professionals.

## Figures and Tables

**Table 1:** Characteristics of the 129 videos.

Characteristics	Frequency, *n* (%)
**Country of publication**	
Mexico	33 (25.6)
Spain	15 (11.6)
Colombia	13 (10.1)
Chile	12 (9.3)
Argentina	11 (8.5)
The United States of America	10 (7.8)
Peru	7 (5.4)
Venezuela	7 (5.4)
Ecuador	6 (4.7)
Guatemala	4 (3.1)
Others	11 (8.5)
**Type of authorship**	
Mass media	73 (56.6)
Health professionals	28 (21.7)
Private users	19 (14.7)
Others	9 (7.0)
**Recommendation according to the WHO**	
Wash your hands frequently	92 (71.3)
Respiratory hygiene	53 (41.1)
Social distance	52 (40.3)
Avoid touching eyes, nose, and mouth	31 (24.0)

**Table 2:** Recommendations to prevent transmission of coronavirus disease 2019 (COVID-19) according to the authorship of the video.

Recommendation	Type of Authorship	Available, *n* (%)	Unavailable, *n* (%)	OR (95% CI) ^a^	*p*
Wash your hands frequently	Health professionals	25 (27.2)	3 (8.1)	5.19 (1.43–18.78)	0.007
	Private users	15 (16.3)	4 (10.8)	2.33 (0.70–7.74)	0.161
	Others	7 (7.6)	2 (5.4)	2.18 (0.42–11.24)	0.346
	Mass media	45 (48.9)	28 (75.7)	1	
Respiratory hygiene	Health professionals	18 (34.0)	10 (13.1)	4.46 (1.77–11.23)	0.001
	Private users	9 (17.0)	10 (13.2)	2.23 (0.79–6.26)	0.126
	Others	5 (9.4)	4 (5.3)	3.09 (0.76–12.67)	0.105
	Mass media	21 (39.6)	52 (68.4)	1	
Social distance	Health professionals	11 (21.1)	17 (22.1)	0.93 (0.38–2.26)	0.869
	Private users	9 (17.3)	10 (13.0)	1.29 (0.47–3.56)	0.624
	Others	2 (3.9)	7 (9.1)	0.41 (0.08–2.11)	0.276
	Mass media	30 (57.7)	43 (55.8)	1	
Avoid touching eyes, nose, and mouth	Health professionals	12 (38.7)	16 (16.3)	4.73 (1.73–12.88)	0.002
	Private users	6 (19.4)	13 (13.3)	2.91 (0.89–9.42)	0.069
	Others	3 (9.7)	6 (6.1)	3.15 (0.68–14.67)	0.131
	Mass media	10 (32.2)	63 (64.3)	1	

^a^ OR (95% CI): Odds Ratio (95% confidence interval).

**Table 3:** Recommendations to prevent transmission of COVID-19 according to the type of transmission of COVID-19 in the country of the video.

Recommendation	Country of Publication	Available, *n* (%)	Unavailable, *n* (%)	OR (95% CI) ^a^	*p*
Wash your hands frequently	Countries with local transmission ^b^	63 (68.5)	18 (48.6)	2.29 (1.05–5.01)	0.036
	Countries with only imported cases ^c^ and countries without cases ^d^	29 (31.5)	19 (51.4)	1	
Respiratory hygiene	Countries with local transmission ^b^	39 (73.6)	42 (55.3)	2.26 (1.06–4.82)	0.035
	Countries with only imported cases ^c^ and countries without cases ^d^	14 (26.4)	34 (44.7)	1	
Social distance	Countries with local transmission ^b^	37 (71.2)	44 (57.1)	1.85 (0.87–3.92)	0.108
	Countries with only imported cases ^c^ and countries without cases ^d^	15 (28.8)	33 (42.9)	1	
Avoid touching eyes, nose, and mouth	Countries with local transmission ^b^	22 (70.9)	59 (60.2)	1.62 (0.67–3.88)	0.282
	Countries with only imported cases ^c^ and countries without cases ^d^	9 (29.1)	39 (39.8)	1	

^a^ OR (95% CI): Odds Ratio (95% confidence interval). ^b^ Countries with local transmission: Spain, the United States of America, Argentina, Colombia, Ecuador, Germany, Chile, Peru, Costa Rica, Dominican Republic, China, the United Kingdom.^c^ Countries with only imported cases: Mexico, Venezuela, Guatemala, Bolivia. ^d^ Countries without cases: Nicaragua, El Salvador.
